# Deciphering the Principles of Bacterial Nitrogen Dietary Preferences: a Strategy for Nutrient Containment

**DOI:** 10.1128/mBio.00792-16

**Published:** 2016-07-19

**Authors:** Jilong Wang, Dalai Yan, Ray Dixon, Yi-Ping Wang

**Affiliations:** a State Key Laboratory of Protein and Plant Gene Research, School of Life Sciences, Peking University, Beijing, China; b School of Advanced Agricultural Sciences, Peking University, Beijing, China; c Department of Microbiology and Immunology, Indiana University School of Medicine, Indianapolis, Indiana, USA; d Department of Molecular Microbiology, John Innes Centre, Norwich, United Kingdom

## Abstract

A fundamental question in microbial physiology concerns why organisms prefer certain nutrients to others. For example, among different nitrogen sources, ammonium is the preferred nitrogen source, supporting fast growth, whereas alternative nitrogen sources, such as certain amino acids, are considered to be poor nitrogen sources, supporting much slower exponential growth. However, the physiological/regulatory logic behind such nitrogen dietary choices remains elusive. In this study, by engineering *Escherichia coli*, we switched the dietary preferences toward amino acids, with growth rates equivalent to that of the wild-type strain grown on ammonia. However, when the engineered strain was cultured together with wild-type *E. coli*, this growth advantage was diminished as a consequence of ammonium leakage from the transport-and-catabolism (TC)-enhanced (TCE) cells, which are preferentially utilized by wild-type bacteria. Our results reveal that the nitrogen regulatory (Ntr) system fine tunes the expression of amino acid transport and catabolism components to match the flux through the ammonia assimilation pathway such that essential nutrients are retained, but, as a consequence, the fast growth rate on amino acids is sacrificed.

## IMPORTANCE

Bacteria exhibit different growth rates under various nutrient conditions. These environmentally related behaviors reflect the coordination between metabolism and the underlying regulatory networks. In the present study, we investigated the intertwined nitrogen metabolic and nitrogen regulatory systems to understand the growth differences between rich and poor nitrogen sources. Although maximal growth rate is considered to be evolutionarily advantageous for bacteria (as remarked by François Jacob, who said that the “dream” of every cell is to become two cells), we showed that negative-feedback loops in the regulatory system inhibit growth rates on amino acids. We demonstrated that in the absence of regulatory feedback, amino acids are capable of supporting fast growth rates, but this results in ammonia leaking out from cells as “waste,” benefiting the growth of competitors. These findings provide important insights into the regulatory logic that controls metabolic flux and ensures nutrient containment but consequently sacrifices growth rate.

## INTRODUCTION

Previous physiological studies demonstrated that glutamine serves as an internal sensor of external nitrogen availability in enteric bacteria (1, 2). Under nitrogen-limiting conditions, the bacterial nitrogen regulatory (Ntr) system responds to the decrease in the internal concentration of glutamine and activates the expression of Ntr-regulated genes/operons (3–5), whose products facilitate the efforts of bacteria to scavenge nitrogenous compounds available in the environment (6).

The Ntr system of *Escherichia coli* comprises a hierarchical regulatory network, including the bifunctional uridylyltransferase/uridylyl-removing enzyme (UTase/UR) GlnD, the two PII signal transduction proteins GlnB and GlnK, and the NtrBC two-component regulatory system (7–11). Under nitrogen-limiting conditions, GlnD covalently modifies GlnB, enabling NtrB to phosphorylate NtrC, which then activates Ntr-dependent genes, including the expression of *glnK* (which encodes GlnK). Although GlnD also covalently modifies GlnK under nitrogen-deficient conditions, there is evidence that GlnK feedback inhibits some Ntr-dependent promoters during nitrogen starvation (5), which is likely to result from the incomplete uridylylation of GlnK. This interaction of the non-covalently modified form of GlnK with NtrB represses the kinase activity and activates the phosphatase activity of NtrB (12). The consequent dephosphorylation of NtrC prevents activation of Ntr-dependent genes. However, the physiological function of GlnK during nitrogen-limiting exponential growth is unclear.

Growth rate maximization is considered to be an important factor in the survival and fitness of unicellular organisms. Among various nitrogen sources, bacteria prefer ammonia, which supports a fast growth rate in *E. coli* compared with alternative nitrogen sources such as amino acids (13). It has been confirmed that the Ntr system maintains a fast growth rate across a wide range of ammonium concentrations, primarily by regulating both the expression and activity of the glutamine synthesis enzyme GS (glutamine synthetase) and also of the ammonium transporter AmtB (5, 14–16).

Alternative nitrogen sources, such as amino acids, support much slower growth rates and are considered to be “poor” nitrogen sources (1), although genes for the utilization of certain amino acids also belong to the Ntr regulon. This nitrogen preference is likely to reflect physico-chemical constraints on the transport and catabolism of amino acids. However, our studies suggest that this nitrogen dietary preference is deliberately maintained by regulatory constraints, enforced particularly by the Ntr system.

We report a direct correlation between nitrogen influx (*J_N_*) and growth rate in *E. coli*, regardless of the nitrogen source used, and demonstrate that the slow growth rate on specific amino acids is limited by constraints on transport and catabolism, as anticipated (17). In contrast, when two transport-and-catabolism (TC)-enhanced (TCE) strains were constructed, they exhibited fast growth on cognate amino acids, comparable to the growth rates observed with ammonium as the sole nitrogen source. Remarkably, these engineered strains prefer to utilize the cognate amino acid rather than ammonia as the sole nitrogen source. However, this switch in nitrogen dietary preferences results in ammonium leakage from the TCE strains, benefiting the growth of competitors. Our quantitative analysis demonstrated that in wild-type *E. coli*, nutrient leakage is efficiently prevented by two negative-feedback loops in the Ntr system that downregulate the expression of the TC genes, which, in turn, determines the slow growth rate on amino acids.

## RESULTS

### Linear relationship between the *J_N_* and growth rate.

**FIG 1:**
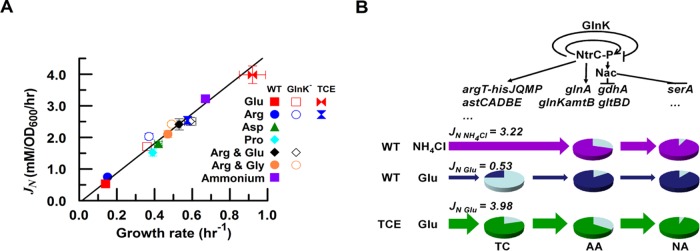
Influence of the nitrogen source on the growth rate of wild-type (WT) and engineered *E. coli* strains. (A) Linear relationship between growth rate and total nitrogen influx (*J_N_*). Data are expressed as means ± standard deviations (SD). (B) Metabolism-regulation model. The upper panel shows the regulatory logic for nitrogen source utilization. The lower panel shows the metabolic logic for utilization of ammonium or amino acids as a nitrogen source. Amino acid metabolism is sequentially divided into three components: amino acid transport and catabolism (TC), ammonium assimilation (AA), and nitrogen anabolism (NA). To illustrate the metabolic capabilities of the TC, AA, and NA components, the coarse-grained model is represented in the form of pie charts. The dark-colored part of each pie represents the maximal metabolic capability of each component in strains grown with the specified nitrogen sources, qualitatively estimated from the expression levels of the corresponding metabolic genes/operons controlled by the regulatory system indicated in the upper panel. The growth phenotypes under different nitrogen conditions are coordinately determined by the metabolic capabilities of the TC, AA, and NA components. The wild-type (WT) strain exhibited a fast growth rate (high *J_N NH4Cl_*) on ammonium due to the high metabolic capabilities of the AA and NA components (represented by the dark pink in the AA and NA pie charts). However, the WT strain exhibited a slow growth rate (low *J_N Glu_*) on glutamate due to the limited metabolic capabilities of the TC component (represented by the dark blue in the TC pie chart), although the metabolic capability of the AA component under these conditions was even higher than that seen under ammonium excess conditions. The glutamate-transport-and-catabolism-enhanced (TCE-Glu) strain exhibited a fast growth rate on glutamate due to the enhanced metabolic capabilities of the TC component (represented by the dark green in the TC pie chart). Although the maximal metabolic capability of the AA component of the TCE-Glu strain grown on Glu was lower than that of the wild-type strain grown under these conditions, it was similar to that of the wild-type strain grown under conditions of excess ammonium, which support a fast growth rate. Therefore, it is not surprising that the presence of the AA component of the TCE-Glu strain is sufficient to support fast growth on Glu.

Consistent with previous data for *Salmonella enterica* serovar Typhimurium (1), ammonium supported the fastest growth rate of *E. coli*, whereas much slower growth rates were observed when individual amino acids were used as the sole nitrogen source (Table 1). To investigate the relationship between growth rate and nitrogen metabolism, we measured the total nitrogen influx (*J_N_*) during cell growth on different nitrogen sources (Table 1; see Materials and Methods and Fig. S1 in the supplemental material for the calculation of the *J_N_* value). A linear relationship between *J_N_* and growth rate was observed (Fig. 1A), thus confirming that growth rate is quantitatively dependent on the rate of nitrogen utilization.

**TABLE 1:** Growth rates and total nitrogen influx measurements

Strain^a^	Nitrogen source	μ (h^−1^)	*J_N_* (mM/OD_600_/h)^b^
WT	Ammonium	0.67 ± 0.02	3.22 ± 0.07
	Glutamate	0.16 ± 0.01	0.53 ± 0.03
	Arginine	0.16 ± 0.01	0.75 ± 0.02
GlnK^−^	Glutamate	0.36 ± 0.00	1.71 ± 0.06
	Arginine	0.37 ± 0.02	2.03 ± 0.09
TCE-Glu	Glutamate	0.85 ± 0.04	3.98 ± 0.28
TCE-Arg	Arginine	0.58 ± 0.04	2.54 ± 0.13

a WT, GlnK^−^, TCE-Glu, and TCE-Arg represent the wild-type (PKUW13), *glnK* (PKUW23), glutamate TCE (PKUW151), and arginine TCE (PKUW81) strains, respectively. μ, growth rate; *J_N_*, total nitrogen influx.

b See Materials and Methods and Fig. S1 in the supplemental material for the calculation of total nitrogen influx (*J_N_*). Data are indicated as means ± SD.

### The nitrogen metabolism-regulation model.

On the basis of the linear growth rate relationship shown in Fig. 1A, we propose that the phenotype of slow growth on alternative nitrogen sources relates to the level of *J_N_*, as depicted in the metabolism-regulation model (Fig. 1B). The Ntr system regulates both amino acid metabolism and ammonium assimilation (AA) in *E. coli* by controlling the expression of some transport and metabolic genes/operons (Fig. 1B, upper panel), and it is well accepted that transport and catabolism (TC) of amino acids generate ammonium and/or glutamate (13). These metabolites enter the AA process to enable glutamine biosynthesis, catalyzed by GS. The products of ammonium assimilation, glutamine and glutamate, provide nitrogen for almost all the nitrogenous compounds assimilated (i.e., by nitrogen anabolism [NA]) in the cell (Fig. 1B, lower panel). The TC components are upstream of the AA pathway in the metabolic architecture; therefore, compared to the total nitrogen influx of the wild-type strain on ammonium (*J*_N NH4Cl_ = 3.22), the low levels of nitrogen influx on arginine (*J*_N Arg_ = 0.75) or glutamate (*J*_N Glu_ = 0.53) are likely to be limited by the TC components, since utilization of either ammonium or amino acids as nitrogen sources involves the same AA and NA components (Fig. 1B).

### TCE strains exhibit fast growth on amino acids.

To verify this hypothesis, we constructed amino acid transport-and-catabolism-enhanced (TCE) strains for the utilization of either glutamate (TCE-Glu) or arginine (TCE-Arg) as the sole nitrogen source (see Materials and Methods). In the presence of saturating concentrations (≥40 µM) of IPTG (isopropyl-β-d-thiogalactopyranoside), the enhanced expression of the TC components increased *J_N_* as well as the growth rate of each TCE strain on the cognate amino acid (Table 1 and Fig. 1). In the case of the TCE-Glu strain, glutamate supported a growth rate (0.85 h^−1^) faster than that of the wild-type strain grown on ammonium (0.67 h^−1^). Although arginine degradation should release more ammonium per amino acid, the growth rate of the TCE-Arg strain (0.58 h^−1^) was notably slower than that of the TCE-Glu strain. Kinetic limitations in either the transport or catabolism components of the TCE-Arg strain may account for this difference. These results confirm that the growth rate on amino acids is limited by *J_N_*, which in turn is limited by the TC components, irrespective of the nature of the nitrogen source.

### Altered nitrogen diet of the TCE strains.

**FIG 2:**
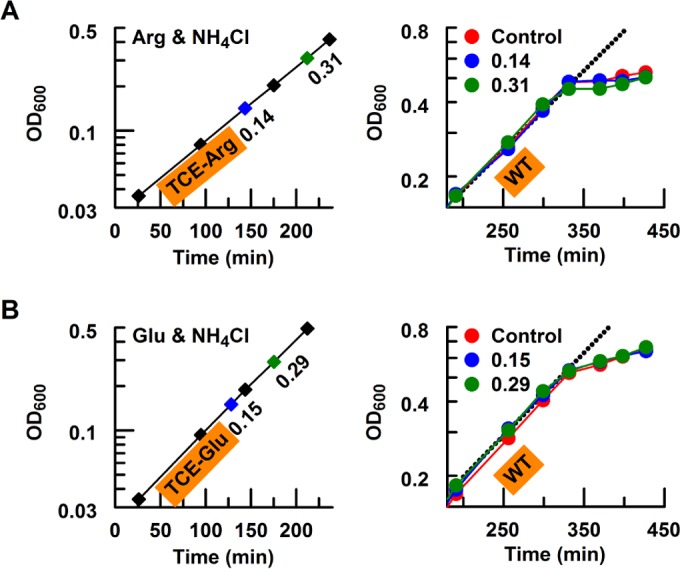
Overexpression of the TC components alters nitrogen dietary preference. (A) Altered nitrogen diet of the TCE-Arg strain. The TCE-Arg strain (PKUW81) was initially grown on 5 mM arginine and 2 mM ammonium (Arg and NH_4_Cl; left panel). At the indicated OD_600_ values, samples of the culture medium were collected by filtration through a 0.22-µM-pore-size filter. The filtrate of the culture medium was then used for growth of the wild-type strain (PKUW13; right panel). In all cases, the wild-type strain grew at an initial rate similar to that of the TCE strain but exhibited a diauxic growth pattern, which we interpret as having been a consequence of switching to the amino acid as the nitrogen source, following depletion of ammonium in the culture medium. The different colors represent culture medium filtrates taken at the corresponding OD_600_ points, while red circles represent wild-type cultures grown on fresh medium (i.e., in the absence of TCE culture filtrates). (B) Altered nitrogen diet of the TCE-Glu strain. The conditions used for this experiment were similar to those described for panel A, except that 5 mM arginine was replaced by 20 mM glutamate (Glu and NH_4_Cl) and culture filtrates of the TCE-Glu strain (PKUW151; left panel) were used for growth of wild-type bacteria (right panel). All media were supplemented with 40 µM IPTG.

Compared to the TCE strains, the wild-type strain does not exhibit maximal growth rates on amino acids, suggesting that fast growth may be not the primary criterion for the metabolism of alternative nitrogen sources. Surprisingly, the improved utilization of the cognate amino acid suppressed the assimilation of ammonium in the TCE strains (Fig. 2). When grown on a mixture of ammonium and amino acids as nitrogen sources, the wild-type strain displayed diauxic growth, exhibiting a preference to utilize ammonium rather than the amino acid (see Fig. S2 in the supplemental material). To investigate the nitrogen source priority in TCE strains, we investigated which nitrogen sources were made available to the wild type in culture filtrates of the TCE strain. Initially, TCE strains were grown on cognate amino acids supplemented with 2 mM ammonium and filtered samples of the culture medium were taken during exponential growth (Fig. 2, left panels). The wild-type strain was then grown in this filtered medium derived from the TCE strains (Fig. 2, right panels). In all cases, we observed that the wild-type strain exhibited diauxic growth on this combination of nitrogen sources, irrespective of whether it was grown in fresh medium as a control (Fig. 2, red circles) or in filtered medium derived from the TCE strains (Fig. 2, blue and green circles). This suggests that TCE strains metabolized their cognate amino acid first, leaving the ammonium level in the medium almost unchanged. Thus, the newly constructed TCE strains had switched to an alternative nitrogen diet, with a growth advantage on the cognate amino acids.

### Cost/benefit analysis of TCE strains.

It is intriguing that *E. coli* is not evolutionary optimized with respect to the utilization of amino acids as nitrogen sources. We evaluated the cost/benefit for TCE strains on different nitrogen sources by calculating the reductions or increases in growth rate relative to the growth rate of the wild-type strain on 20 mM ammonium (see Materials and Methods). The benefit of removing all native regulatory constraints on glutamate transport is that it confers a growth advantage of 0.27 for the TCE-Glu strain (PKUW151) when induced with saturating concentrations of IPTG (see Table S1 in the supplemental material). However, optimal expression of the TC genes/operon for the amino acid may itself incur a cost penalty associated with increased protein expression (18, 19). To assess the cost of expressing the arginine TC enzymes, we measured the growth rates of the TCE-Arg strain (PKUW81) grown on ammonium at various IPTG concentrations. Since there is no benefit in expressing these components when strains are grown on ammonium, any growth rate penalties reflect the costs of TC operon expression. Under these conditions, we observed a linear cost dependence on the expression of these operons, representing a 5% cost burden for cells upon full induction of the TC components with IPTG (see Fig. S3 in the supplemental material). Thus, the benefits of optimal TC expression far outweigh the costs of the increased protein synthesis required.

### The TCE-Arg strain is oversensitive to the toxic amino acid analogue l-canavanine.

Increasing the efficiency of amino acid transport in TCE strains may incur risks associated with an enhanced ability to import toxic amino acid analogs (20), which may counterbalance the growth advantage of TCE strains. To investigate this, l-canavanine, a toxic analogue of arginine produced by leguminous plants (21–24), was added to cultures of the TCE-Arg strain (PKUW81) grown in minimal medium containing 20 mM ammonium as the nitrogen source and induced with 40 µM IPTG (see Fig. S4 in the supplemental material). In the absence of l-canavanine, the TCE-Arg strain and the wild-type strain had similar growth rates. In contrast, growth of the TCE-Arg strain was far more sensitive to the presence of l-canavanine than growth of the wild-type strain. The TCE-Glu strain (PKUW151) was also tested and behaved similarly to the wild-type strain under these conditions, indicating that the effect observed with the TCE-Arg was due to the toxic effects of l-canavanine transported specifically through the overexpressed arginine transport system. Thus, TCE strains may exhibit increased sensitivity to toxic analogs in the natural environment.

### Nutrient leakage from TCE strains.

**FIG 3:**
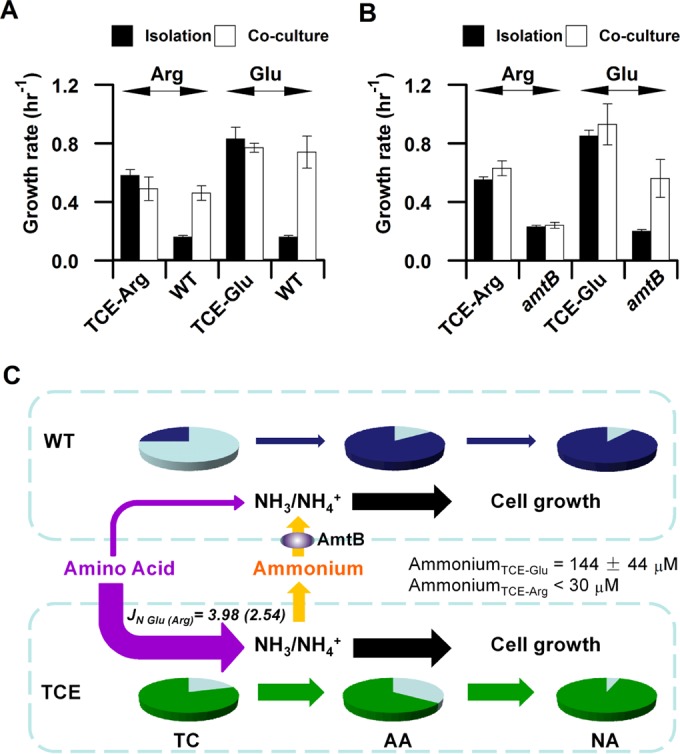
Nutrient leakage in TCE strains. (A) Relative fitness levels of the TCE strains cocultured with the wild-type strain. In each case, the indicated TCE strain and its wild-type counterpart were grown either in isolation or in coculture using the same growth media. The growth status of each TCE strain (kanamycin resistant [Kan^r^]) or of its cocultured wild-type counterpart (Kan^s^) was determined in the coculture by plate counting on LB medium in the presence or absence of kanamycin. Arg, TCE-Arg strain (PKUW81) and wild-type strain (PKUW33) grown on 5 mM arginine plus 40 µM IPTG; Glu, TCE-Glu (PKUW151) and wild-type strains grown on 20 mM glutamate plus 40 µM IPTG. (B) Relative fitness levels of the TCE strains (Kan^r^) cocultured with the *amtB* strain (PKUW36; Kan^s^). The growth media and conditions were the same as those described for panel A. (C) Nutrient containment model. The pie charts are represented as described in the Fig. 1B legend. The limited metabolic capability of the TC component (represented by dark blue in the TC pie chart) repressed the utilization of amino acids in the wild-type strain. When TCE cells were cocultivated with the wild-type strain and the cognate amino acid was used as the sole nitrogen source, enhanced TC component expression (represented by the dark green in the TC pie chart) in the TCE strains converted the cognate amino acid into ammonium more efficiently. As a consequence of the high internal ammonium concentration, ammonium was excreted into the medium, which in turn was used by the cocultivated wild-type bacteria as the preferred nitrogen source, compensating for the low amino acid influx and supporting fast growth. AmtB in the wild-type cells is required for scavenging ammonium in the medium. “Ammonium_TCE-Glu_” (or “Ammonium_TCE-Arg_)” represents ammonium leakage from TCE strains, with the cognate amino acid as the nitrogen source. The growth media were the same as those described for panel A. Filtered medium samples were taken at an OD_600_ of approximately 0.4. Data are expressed as means ± SD.

The change in nitrogen dietary preferences resulted in nutrient leakage from the TCE strains, benefiting the growth of competitors (Fig. 3). When arginine (or glutamate) was the sole nitrogen source, cocultivation with the cognate TCE strain enhanced the growth rate of the wild-type strain by ~2.88-fold (or 4.63-fold) (Fig. 3A). However, the growth enhancement was not observed when the TCE-Arg strain was cocultivated with a strain lacking the *amtB* ammonium transporter gene (Fig. 3B), suggesting that ammonium produced by the TC component is excreted from the TCE strain. Although growth enhancement still existed when the TCE-Glu strain was cocultivated with the *amtB* strain, the concentration of ammonia released by this engineered strain was sufficient for passive diffusion rather than activation of ammonium uptake by AmtB (14). Direct measurement of the ammonia concentration in the culture confirmed that the level of ammonia excreted from the TCE-Arg strain was below the threshold required to activate AmtB-mediated ammonium uptake (30 µM) (14), but the TCE-Glu strain released 144 µM ammonia into the medium after 4 h of growth in exponential phase (Fig. 3C). Therefore, the growth advantage of TCE strains on the cognate amino acids was counterbalanced by a deficiency in nutrient containment.

### The Ntr system finely controls the expression of amino acid transport and catabolic genes, in order to retain nutrient.

Nitrogen-limiting conditions are imposed when amino acids are utilized as the sole nitrogen source, signaling the Ntr system to activate the expression of Ntr genes/operons (11, 14, 16). Since the GlnK signal transduction protein feedback inhibits the expression of some Ntr genes during ammonium starvation (5), we investigated whether *glnK* influences growth rates in cultures with amino acids as the sole nitrogen source. Consistent with previous observations in liquid glucose-arginine medium (8), the *glnK* deletion strain exhibited a 2-fold increase in growth rate compared with its wild-type counterpart when either arginine or glutamate was used as the sole nitrogen source (Table 1), suggesting that the wild-type strain maintains relatively slow growth rates on amino acids via exacting regulation by the Ntr system (Fig. 1B).

The metabolism-regulation model shown in Fig. 1B predicts that such growth rate enhancement in the *glnK* strain requires an increase in *J_N_* which could be satisfied by NtrC-P-mediated upregulation of TC operon expression in the absence of negative feedback by GlnK. Accordingly, gene expression levels were compared when arginine was used as the sole nitrogen source. Expression from the arginine transport (*argTp*) and catabolic (*astC*) promoters was increased in the *glnK* strain (Table 2), whereas the *gltB* (encoding the large subunit of glutamate synthase) promoter was repressed by NtrC-P, as anticipated (see Fig. 1B) (25).

**TABLE 2:** Gene expression differences between wild-type and *glnK* strains

Strains	Gene expression ratio^a^					
	*argT*		*astC*		*gltB*	
	qPCR^b^	β-Gal^c^	qPCR	β-Gal	qPCR	β-Gal
GlnK^−^/WT	3.2 ± 1.0	2.4 ± 0.1	1.3 ± 0.2	1.9 ± 0.1	0.2 ± 0.1	0.2 ± 0.0

a Data represent gene expression ratios of *glnK* strain to the wild-type counterpart with 5 mM arginine as the sole nitrogen source.

b qPCR, quantitative real-time PCR analysis.

c For the β-galactosidase assays, we compared the LacZ expression level of the corresponding promoter-*lacZ* fusions in the *glnK* strain with that in its wild-type counterpart as follows: for *argTp-lacZ*, PKUW61 (*argTp-lacZ*) and PKUW63 (*glnK argTp-lacZ*); for *astC-lacZ*, PKUW67 (*astCp-lacZ*) and PKUW68 (*glnK astCp-lacZ*); for *gltBp-lacZ*, PKUW72 (*glnBp-lacZ*) and PKUW64 (*glnK gltBp-lacZ*).

**FIG 4:**
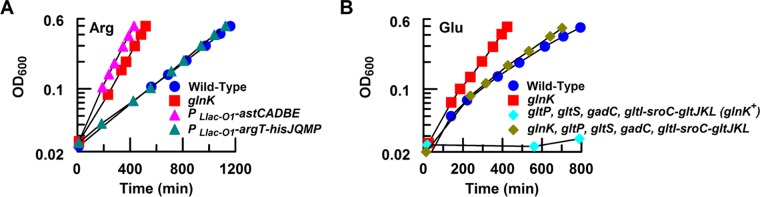
Identification of the genes/operons responsible for growth rate limitation. (A) The limiting step(s) for growth on arginine. Arginine (Arg) (5 mM) was used as the sole nitrogen source, and IPTG (40 µM) was added to induce the expression of the *P_Llac-O1_-argT-hisJQMP* operon (PKUW59; *glnK^+^*; green triangle) or the *P_Llac-O1_-astCADBE* operon (PKUW78; *glnK^+^*; pink triangle). The wild-type (PKUW13; blue circle) and *glnK* (PKUW23; red square) strains were used as control strains. (B) The limiting step(s) for growth on glutamate. Glutamate (Glu) (20 mM) was used as the sole nitrogen source. Without the four known glutamate transport systems, the *gltP gltS gadC gltI-sroC-gltJKL* strain (PKUW195; *glnK^+^*; cyan diamond) had a growth defect. However, the *glnK gltP gltS gadC gltI-sroC-gltJKL* strain (PKUW196; *glnK* deletion; brown diamond) exhibited a growth rate (0.19 h^−1^) similar to that of the wild-type strain (PKUW13; blue circle).

In order to further test which operon(s) contributes to the GlnK-mediated growth inhibition, we genetically manipulated amino acid transport and catabolic operons separately. We replaced the native promoters for the arginine transport operon *argT-hisJQMP* and catabolic operon *astCADBE* with the synthetic *P_Llac-O1_* promoter (26, 27), hence disrupting their regulatory relationship with GlnK and enabling controlled expression by the inducer IPTG. When the *argT* transporter was expressed from the *P_Llac-O1_* promoter with 40 µM IPTG (strain PKUW59; *glnK*^+^), the growth rate on arginine was similar to that of the wild-type strain (Fig. 4A), indicating that the overexpression of this transporter does not relieve GlnK-mediated growth inhibition (Table 1). In contrast, overexpression of the *astCADBE* operon in the *glnK*^+^ background (strain PKUW78) resulted in an increased growth rate on arginine similar to that of the *glnK* deletion strain (Fig. 4A), indicating that the negative influence on growth rate imposed by GlnK can be bypassed through NtrC-independent expression of the arginine catabolic operon.

To investigate which glutamate transport system(s) might be responsible for GlnK-dependent growth inhibition in the presence of glutamate (Table 1), we deleted glutamate transport systems and examined growth rates. When all four known glutamate transport systems (*gltP*, *gltS*, *gadC*, *gltI-sroC-gltJKL*) were deleted, the mutant strain (PKUW195; *glnK*^+^) was unable to grow on glutamate, as anticipated (Fig. 4B). However, to our surprise, when the *glnK* gene was also deleted in the *gltP*, *gltS*, *gadC*, *gltI-sroC-gltJKL* background (strain PKUW196; *glnK* deletion), the ability to grow on glutamate was restored, with a growth rate similar to that of the wild-type strain (PKUW13) (Fig. 4B). This suggests that there is an unknown transport system(s) that can functionally transport glutamate but this transporter is inhibited by the presence of GlnK.

These results demonstrate that the presence of GlnK results in repression of the catabolic operon for arginine (*astCADBE*) and of an unknown transport gene(s) for glutamate, ensuring fine tuning of nitrogen influx and growth rate on these alternative nitrogen sources. Similarly to TCE strains, the growth advantage of the *glnK* deletion strain was also counterbalanced by the deficiency in nutrient containment (Table 3).

**TABLE 3:** Relative fitness of the *glnK* strain (PKUW38; Kan^s^) cocultured with the wild-type strain (PKUW13; Kan^r^)

Strain^a^	Arginine		Glutamate	
	μ (h^−1^)	*R^b^*	μ (h^−1^)	*R*
Mixture^c^	0.31 ± 0.00		0.36 ± 0.03	
*glnK*_C_^d^	0.34 ± 0.00	−0.08	0.40 ± 0.04	0
*glnK*_S_^e^	0.37 ± 0.02		0.40 ± 0.01	
WT_C_	0.28 ± 0.01	0.75	0.32 ± 0.05	0.88
WT_S_	0.16 ± 0.01		0.17 ± 0.00	

a Arginine (5 mM) (arginine columns) or glutamate (20 mM) (glutamate columns) was used as the sole nitrogen source. Data are expressed as means ± SD.

b *R* = (μ_C_ − μ_S_)/μ_S_, where μ_C_ and μ_S_ represent the growth rates of the same strain in the coculture and in isolation, respectively.

c Data represent the total growth rate of the coculture.

d “*glnK*_C_” or “WT_C_” data represent the deduced growth rates of the *glnK* or wild-type strain in the coculture, respectively.

e “*glnK*_S_” and “WT_S_” data represent the growth rates of the *glnK* and wild-type strain grown in isolation.

**FIG 5:**
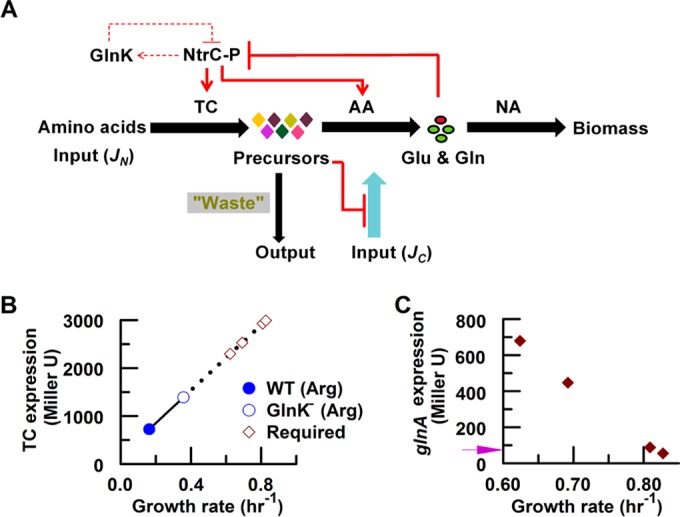
The nitrogen regulatory system finely controls the expression of amino acid transport and catabolic genes to retain nutrient. (A) Model for Ntr-mediated nutrient retention. Solid and dashed red lines/arrows represent different negative-feedback loops. For the TCE-Glu strain grown on glutamate, the carbon skeleton derived from the amino acid (e.g., α-ketoglutarate) represses the carbon influx (*J_C_*; aqua arrow) through carbon catabolite repression. (B) Relationship between growth rate (and *J_N_*) and TC expression, with arginine used as the sole nitrogen source. The β-galactosidase activity of an *astC-lacZ* fusion was used to monitor expression of the NtrC-dependent *astCADBE* operon in the wild-type strain (PKUW67; closed blue circle) and the *glnK* strain (PKUW68; open blue circle). Open diamonds represent the predicted expression levels of the *astCADBE* operon in the wild-type strain required to achieve the same growth rate on arginine as that of the TCE-Glu strain grown on glutamate (see panel C). (C) Ntr-dependent activation of the *glnA* promoter was reduced in the TCE-Glu strain as the growth rate increased. The TCE-Glu strain (PKUW218) was grown on 20 mM glutamate supplemented with different concentrations of IPTG. The violet arrow represents wild-type *E. coli* (NQ158) grown on 20 mM ammonium.

Although deletion of GlnK increased the expression of the TC components and enhanced bacterial growth (Fig. 5A), the growth rate of the *glnK* deletion strain was much slower than that of the TCE strains on the cognate amino acids (Table 1). For example, although the expression of the catabolic operon for arginine (*astCADBE*) was increased by ~2-fold in the *glnK* strain, with a corresponding increase in *J_N_*, we calculate that the expression level of this operon would have to increase to at least 3-fold higher than that observed with the wild-type strain (open diamonds in Fig. 5B) in order to achieve the fast growth rates exhibited by the TCE-Glu strain (Fig. 5C). Previous physiological studies demonstrated that glutamine serves as an internal sensor of external nitrogen availability in enteric bacteria (1, 2). Regardless of whether the native strength of TC promoters can support this level of expression, glutamine-mediated negative feedback in the Ntr system downregulated the expression of the Ntr genes/operons (including *astCADBE*) as the growth rate increased. Using the *glnA* (glutamine synthetase) promoter as a monitor of Ntr-mediated regulation, we observed a reciprocal relationship between growth rate and the expression of *glnA* when an IPTG concentration gradient was applied to enable determination of the titers resulting from expression of the glutamate transporters in the TCE-Glu strain (Fig. 5C). Thus, although Ntr-mediated regulation of the TC components was circumvented in the TCE strains, the AA pathway remained subject to nitrogen regulation (Fig. 5A). As the internal glutamate concentration is likely to be saturating in the fully induced TCE-Glu strain, substrate availability for GS is likely to be nonlimiting, resulting in a high rate of glutamine biosynthesis and consequent feedback inhibition of the Ntr system. Downregulation of GS under these conditions is similar to the response of the wild-type strain under ammonium excess conditions. When the internal ammonia concentration in TCE-Glu cells exceeds the capacity for assimilation by GS, ammonia leaks out of the cells by passive diffusion across the cell membrane. The decreased ability of nonmodified NtrC to activate transcription under these conditions results in decreased expression of the *glnK*-*amtB* operon and, coupled with GlnK-mediated inactivation of AmtB, impedes active transport of ammonium, thus exacerbating the potential for ammonia leakage.

In contrast, in the wild-type strain, the primary restriction on fast growth of cultures grown on alternative nitrogen sources was the high level of NtrC-P required to activate TC expression (Fig. 5B), which in turn was regulated by the negative-feedback loops within the Ntr system that control the NtrC-P level (Fig. 5A and C). Consequently, when the wild-type strain was grown on alternative nitrogen sources, the status of nitrogen limitation was relieved, but not overcome, and the flux through the ammonium assimilation pathway was carefully controlled (Fig. 1B). This ensured close coupling of amino acid transport and catabolism with ammonia assimilation, to prevent nutrient leakage, but sacrificed fast growth rates.

Notably, for the TCE-Glu strain, using glutamate as a nitrogen source also added an additional carbon source, which in turn could affect carbon metabolism. The additional input of carbon skeletons provided by the enhancement of glutamate transport and catabolism in TCE-Glu cells probably also affected the intracellular carbon status reflected by the status of carbon metabolic signal cyclic AMP (cAMP), since 2-oxoglutarate derived from the transamination of glutamate directly inhibits cAMP synthesis. To confirm this, cAMP receptor protein-cAMP complex (CRP-cAMP)-dependent activation of the *lac* system was employed as a monitor of cAMP levels. Indeed, we observed that cAMP signaling was downregulated in the TCE-Glu strain grown on glutamate (see Fig. S5 in the supplemental material). Thus, carbon catabolite repression (CCR) may also influence growth rate (27) under these conditions by modulating the carbon flux (*J_C_*) (Fig. 5A).

## DISCUSSION

The overall strategy for Ntr-mediated regulation of nitrogen assimilation is apparently to optimize the growth rate in relation to internal ammonium availability as determined by the glutamine concentration (1). Our study results highlight the importance of feedback loops mediated by both GlnK and the product (glutamine) in restricting the assimilation of alternative nitrogen sources. Although this has consequences for growth rate and optimal production of biomass, it fine tunes the level of nitrogen metabolites to prevent nutrient leakage. Therefore, we propose that the principle behind the preference for the nitrogen diet is that of trading off the benefits of fast growth on alternative nitrogen sources with the fitness penalty associated with excreting nitrogen that becomes available to competitors. This tradeoff is achieved through the highly complex Ntr regulatory circuitry, which prioritizes nutrient retention over fast growth, resulting in metabolic slowdown on alternative nitrogen sources. It was reported recently that when an alternative carbon source, such as glycerol, is used as the sole carbon source, enzymatic constraints on carbon uptake prevent acetate leakage from wild-type *E. coli* and, as a consequence, result in sacrifice of the enhanced growth rate (28). Therefore, nutrient containment could be a common strategy for both carbon and nitrogen metabolism in *E. coli*.

From the application perspective, it should be feasible to metabolically engineer efficient utilization of other amino acids as nitrogen sources, using synthetic biology approaches similar to those described here. Our studies are thus likely to underpin more-efficient biorefinery processes that utilize waste amino acids as raw material to achieve sustainable biofuel or biochemical production and recover fertilizer nitrogen (29, 30).

## MATERIALS AND METHODS

### Strain construction and growth conditions.

All strains used here (Table 4) were derivatives of *E. coli* K-12 prototrophic strain NCM3722 (31). Mutant strains were constructed by lambda Red-mediated homologous recombination (27, 32) and P1 transduction. For TCE-Glu (PKUW151), the promoters of three of the four known glutamate transport operon/genes (*gltI-sroC-gltJKL*, *gltP*, and *gltS*) were replaced by the IPTG-inducible P_Llac-O1_ promoter (26), and for TCE-Arg (PKUW81), the promoters of both the arginine transport and the arginine catabolic operons (*argT-hisJQMP* and *astCADBE*, respectively) were replaced by the synthetic P_Llac-O1_ promoter.

**TABLE 4:** Strains used in this study

Strain^a^	Genotype
PKUW13	Wild-type (Km^r^)^b^
PKUW15	*ΔglnK*-*amtB* (Km^r^)^b^
PKUW19	*ΔamtB* (Km^r^)^b^
PKUW23	*ΔglnK* (Km^r^)^b^
PKUW33	Wild-type (Km^s^)^c^
PKUW36	*ΔamtB* (Km^s^)^c^
PKUW38	*ΔglnK* (Km^s^)^c^
PKUW59	*attB*::*sp*^r^*-lacI*^q^*-tetR ΔargTp*::*P_Llac-O1_* (Km^r^)^d^
PKUW61	*ΔlacI-Plac*::*argTp* (Km^r^)^e^
PKUW63	*ΔglnK ΔlacI-Plac*::*argTp* (Km^r^)^e^
PKUW64	*ΔglnK ΔlacI-Plac*::*gltBp* (Km^r^)^e^
PKUW67	*ΔlacI-Plac*::*astCp* (Km^r^)^e^
PKUW68	*ΔglnK ΔlacI-Plac*::*astCp* (Km^r^)^e^
PKUW72	*ΔlacI-Plac*::*gltBp* (Km^r^)^e^
PKUW78	*attB*::*sp*^r^*-lacI*^q^*-tetR ΔastCp*::*P_Llac-O1_* (Km^r^)^d^
PKUW81 (**TCE-Arg**)	*attB*::*sp*^r^*-lacI*^q^*-tetR ΔargTp*::*P_Llac-O1_*^f^ *ΔastCp*::*P_Llac-O1_* (Km^r^)^d^
PKUW151 (**TCE-Glu**)	*attB*::*sp*^r^*-lacI*^q^*-tetR ΔgltIp*::*P_Llac-O1_*^f^,*ΔgltSp*::*P_Llac-O1_*^f^,*ΔgltPp*::*P_Llac-O1_* (Km^r^)^d^
PKUW195	*ΔgltS ΔgltP ΔgadC ΔgltI-sroC-gltJKL*::*Kan*^g^
PKUW196	*ΔglnK ΔgltS ΔgltP ΔgadC ΔgltI-sroC-gltJKL*::*Kan*^g^
PKUW218	*attB*::*sp*^r^*-lacI*^q^*-tetR ΔgltIp*::*P_Llac-O1_*^f^ *ΔgltSp*::*P_Llac-O1_*^f^ *ΔgltPp*::*P_Llac-O1_*^f^ *ΔlacI-Plac*::*glnAp* (Km^r^)^e^
NQ158	*ΔlacI-Plac*::*glnAp* (Km^r^)^e,h^

a All strains listed are isogenic with respect to the prototrophic K-12 strain NCM3722. Boldface highlighting indicates TCE strains.

b A DNA fragment containing the kanamycin cassette (from pKD13) followed by the *rrnB* terminator sequence (*rrnBT*) was inserted upstream of the *glnK* promoter (*glnKp*) (27). This DNA fragment (*Kan-rrnBT-glnKp*) was then combined with the wild-type *glnK*-*amtB* operon or different in-frame deletions of the operon (Δ*glnK*, Δ*amtB*, and Δ*glnK*-*amtB*) and then integrated into the chromosome of *E. coli* to replace the *glnK*-*amtB* operon.

c The strain was constructed as described for the strains indicated with the superscript italic “b,” but the kanamycin resistance gene was eliminated by using the helper plasmid pCP20 (32).

d A DNA fragment containing the kanamycin cassette (from pKD13) followed by the *rrnB* terminator sequence (*rrnBT*) was inserted upstream of the *P_Llac-O1_* promoter (26). The DNA fragment (*Kan-rrnBT-P_Llac-O1_*) was then integrated into the chromosome of *E. coli* to replace the promoter region of the target gene. A *sp*^r^-*lacI*^q^-*tetR* cassette providing constitutive expression of *lacI* was inserted at the *attB* site to tightly repress *P_Llac-O1_* activity.

e A DNA fragment containing *Kan-rrnBT* (as described above) and the promoter region of the target gene was integrated into the chromosome of *E. coli* to replace part of *lacI* and the entire P*lacZ* promoter.

f The strain was constructed as described for the strains indicated with the superscript italic “d,” but the kanamycin resistance gene was eliminated by using the helper plasmid pCP20 (32).

g For the in-frame deletion of the operon, a DNA fragment extending from the second codon of the first gene in the operon through to the seventh codon from the C terminus of the last gene in the operon was replaced by the kanamycin cassette (from pKD13).

h This strain was provide by Terence Hwa. All the other strains were constructed in this study.

Since *glnK* and the downstream *amtB* gene are located in the same operon (*glnK*-*amtB*) and controlled at the transcriptional level by a single promoter (*glnKp*), a different strategy was used to construct the *glnK* in-frame deletion strain to prevent polar effects on *amtB*. A kanamycin resistance cassette (from pKD13) followed by the *rrnB* terminator sequence (*rrnBT*) was inserted as a selectable marker upstream of the *glnK* promoter (*glnKp*) (27), combined with an in-frame deletion of *glnK* from codon 2 to codon 106 (the eighth codon from the C terminus) without a scar (32, 33). The recombined DNA fragment was integrated into the chromosome of *E. coli* to replace the *glnKp-glnK* region. The presence of the *rrnB* terminator upstream of the *glnKp* promoter ensured that the kanamycin resistance cassette would not affect the transcription from the *glnKp* promoter. Additional experiments were carried out to exclude polar effects on the downstream *amtB* gene (see Table S2 and Text S1 in the supplemental material).

The default minimal medium was N^–^C^–^ salts (17) plus 0.4% (wt/vol) glycerol as the carbon source. Nitrogen sources were specified in different experiments, and their default concentration was 20 mM total N. All batch cultures were grown aerobically at 37°C with shaking at 220 rpm and were inoculated from a preculture grown in the same medium (34).

### Cost/benefit analysis of TCE strains.

Since the wild-type strain exhibits the fastest growth rate under ammonia excess conditions, we compared the growth rates of strains with different nitrogen sources to the growth rate of the wild-type strain on 20 mM NH_4_Cl as the sole source of nitrogen using the following equation:

$$\text{Cost}/\text{Benefit}=(\mu -\mu_{0})/\mu_{0}$$

where μ is the growth rate of the TCE or wild-type strain with different nitrogen sources and μ_0_ is the growth rate of the wild-type strain on 20 mM NH_4_Cl.

### Ammonia assay.

TCE strains (PKUW81 or PKUW151) were grown in minimal medium with the cognate amino acid (5 mM arginine or 20 mM glutamate) as the sole nitrogen source and supplemented with 40 µM IPTG. Samples were collected at an optical density at 600 nm (OD_600_) of approximately 0.4. A 500-µl aliquot of the culture was filtered through a 0.22-µm-pore-size filter and stored at −80°C. The ammonium concentration in the medium was measured with an ammonia assay kit (AA0100; Sigma).

### Differential expression analysis by qPCR.

The wild-type strain (PKUW13) and the *glnK* strain (PKUW23) were grown in minimal medium with 5 mM arginine as the sole nitrogen source. Samples (2 × 10^8^ cells) were collected at an OD_600_ of ~0.4, frozen quickly in liquid nitrogen, and stored at −80°C. Total RNA was extracted by the use of an RNAprep pure Cell/Bacteria kit (Tiangen), and cDNA was obtained using random hexamer primers and a cDNA library construction kit (TaKaRa). Subsequent quantitative real-time PCR (qPCR) was performed by the use of ReaMaster Mix (SYBR green) (Tiangen), and the raw data were analyzed by the use of DART-PCR version 1.0 to evaluate the threshold cycle (*C_T_*) values and the amplification efficiencies (35). Finally, the measured amplification efficiencies were used to calculate the relative gene transcript amounts. All the target transcripts were normalized to 16S rRNA.

### Differential expression analysis by β-galactosidase assay.

We constructed corresponding promoter-*lacZ* fusions for detecting the expression level of target genes/operons. DNA fragments containing the promoter region of the target genes were integrated into the chromosome of *E. coli* to replace part of *lacI* and the entire P*lacZ* promoter. Samples (~0.3 ml of cell culture) were collected and frozen on dry ice. Five samples were collected for each culture (OD_600_ range, 0.15 to 0.5). The β-galactosidase assay was performed at 37°C according to the traditional Miller method (36). The differential rate—i.e., the “LacZ expression level” (quantified in Miller units)—was obtained as described previously (27).

### Calculation of the total nitrogen influx (*J_N_*).

*E. coli* can grow at a constant rate on amino acids as the sole nitrogen source until the amino acid concentration in the medium drops below a certain threshold, at which point the growth slows down (see Fig. S1 in the supplemental material) (16, 37). For a constant growth rate, we can calculate how much amino acid is needed to provide a certain cell density (OD_600_ = 1):

(1) $${\left[C\right]}_{\text{i}}-{\left[C\right]}_{\text{T}}=1/Y\times ({\text{OD}}_{\text{f}}-{\text{OD}}_{\text{i}})$$

where [*C*]_i_ denotes the initial concentration of amino acid, OD_i_ is the initial OD_600_ value from the time point of the inoculation of the culture, OD_f_ is the final OD_600_ that *E. coli* can maintain a constant growth rate, and [*C*]_T_ is the threshold value below which *E. coli* cannot grow at a constant rate. [*C*]_T_ may vary according to the amino acid used as the nitrogen source. 1/*Y* represents the amino acid consumption, which relates to how much amino acid is needed for growth at a constant rate at an OD_600_ of 1.

Taking the arginine consumption calculation as an example, we grew the wild-type strain (PKUW33) in default minimal medium with the following different concentrations (quantified in millimoles) of arginine [*C*]_i_: 0.1, 0.2, 0.3, 0.4, and 0.5. The cultures were set at the same initial OD_600_ (see Fig. S1 in the supplemental material). The OD_f_ was determined from the growth curves, and by plotting [*C*]_i_ versus OD_f_, the slope (1/*Y*) represents amino acid consumption.

From equation 1, the amino acid concentration in the medium [*C*] and the OD_600_ of the culture have the following relationship:

(2) $$\left[C]={\left[C\right]}_{\text{i}}-1/Y\times (\text{OD}-{\text{OD}}_{\text{i}}\right)$$

where OD is the OD_600_ of the culture and [*C*]_i_, *Y*, and OD_i_ are as defined for equation 1. From equation 2, we can calculate the total amino acid influx:

(3) $$\frac{d[C]}{\text{OD}\cdot dt}\text{μ}/Y$$

where μ represents the growth rate. The total nitrogen influx is then *n*·μ/*Y* (in millimoles per hour divided by the OD_600_), where *n* is number of nitrogen atoms in the amino acid.

When an amino acid combination was used as the nitrogen source, the growth rate of the wild-type strain was much faster than that seen with either of the individual amino acids. The total nitrogen influx was calculated as follows: 1 amino acid (amino acid A) was added at a high level (total concentration = 10 mM), and the concentration of the other amino acid (amino acid B) was varied. We first calculated the nitrogen influx for amino acid B to maintain the fast growth rate. Subsequently, amino acid B was added at a high level (total concentration = 10 mM), and the concentration of amino acid A was varied to calculate the nitrogen influx for amino acid A. The sum of nitrogen influxes for amino acid A and amino acid B is the total nitrogen influx for the amino acid combination.

## SUPPLEMENTAL MATERIAL

Figure S1  Total nitrogen influx (*J_N_*). We used different concentrations (quantified in millimoles) of arginine as the nitrogen source to grow the wild-type strain (PKUW33). From the growth curve, we could calculate the OD_f_ values corresponding to different arginine concentrations. The initial arginine concentration was plotted against the OD_f_ value in the insertion, and the slope represents the amino acid consumption. Thus, the total nitrogen influx is *n* ⋅ μ/*Y* (quantified as millimoles per hour divided by the OD_600_), where *n* is the number of nitrogen atoms in the amino acid. Download Figure S1, PDF file, 0.1 MB

Figure S2  Nitrogen diauxie. The wild-type strain (PKUW13) was grown in minimal medium with 1.5 ammonium (blue circle) or 1.5 mM ammonium plus 5 mM arginine (red circle) as the nitrogen source. Download Figure S2, PDF file, 0.1 MB

Figure S3  Full induction of the TC components results in a cost burden. (A) The TCE-Arg strain (PKUW81) was grown with 20 mM ammonium as the sole nitrogen source. Different concentrations (quantified in micromoles) of IPTG were supplemented to induce the expression of the arginine TC components. (B) The cost of growth of the TCE-Arg strain (PKUW81) with 20 mM ammonium as the sole nitrogen source. To calculate the cost of growth at different concentrations of IPTG, the wild-type strain (PKUW13) was grown in the same media as the TCE-Arg strain (PKUW81). Although different concentrations of IPTG had little effect on the growth rate of the wild-type strain, to exclude effects other than full induction of the TC components, the growth rate of the TCE strain was divided by the growth rate of the wild-type strain at the corresponding IPTG concentration. This normalized value was used to calculate the full induction cost of the TC components. Download Figure S3, PDF file, 0.1 MB

Figure S4  The TCE-Arg strain is more sensitive to l-canavanine. Both the wild-type strain (WT; closed symbols) and the TCE-Arg strain (open symbols) were grown in minimal medium with 40 µM IPTG and 20 mM NH_4_Cl as the nitrogen source. Different concentrations (0, 0.16, and 20 µg/ml) of l-canavanine were added to the cultures as indicated. Download Figure S4, PDF file, 0.1 MB

Figure S5  cAMP signaling in TCE-Glu and wild-type (WT) strains determined by analyzing the expression of the native *lacZ* gene. To fully deactivate LacI, 1 mM IPTG was added to both the precultures and the growth cultures. Data are expressed as means ± SD. Download Figure S5, PDF file, 0.1 MB

Table S1  Cost/benefit when glutamate was used as the sole nitrogen source. Table S1, PDF file, 0.05 MB

Table S2  Growth rates of strains with arginine as the nitrogen source. Table S2, PDF file, 0.1 MB

Text S1 Supplemental text. Download Text S1, PDF file, 0.1 MB
