# Supplementary figures and images for: Deciphering the Principles of Bacterial Nitrogen Dietary Preferences: a Strategy for Nutrient Containment
Source: mBio. 2016 Jul 19;7(4):e00792-16. doi: 10.1128/mBio.00792-16 (PMC4958250; doi:10.1128/mBio.00792-16)

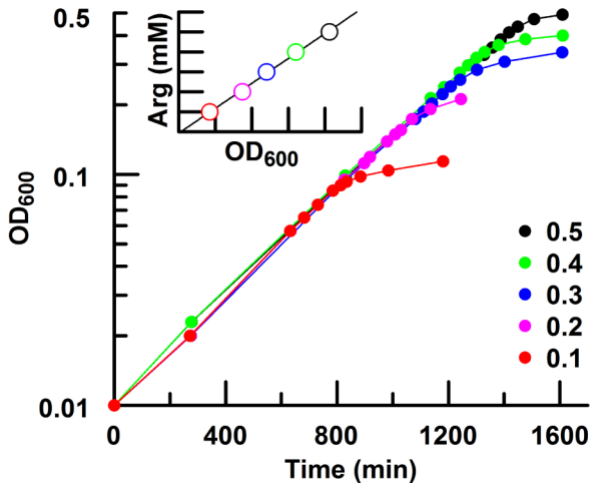

Supplement: Figure S1 — Total nitrogen influx (JN). We used different concentrations (quantified in millimoles) of arginine as the nitrogen source to grow the wild-type strain (PKUW33). From the growth curve, we could calculate the ODf values corresponding to different arginine concentrations. The initial arginine concentration was plotted against the ODf value in the insertion, and the slope represents the amino acid consumption. Thus, the total nitrogen influx is n ⋅ μ/Y (quantified as millimoles per hour divided by the OD600), where n is the number of nitrogen atoms in the amino acid. Download [file mbo004162913sf1.pdf]

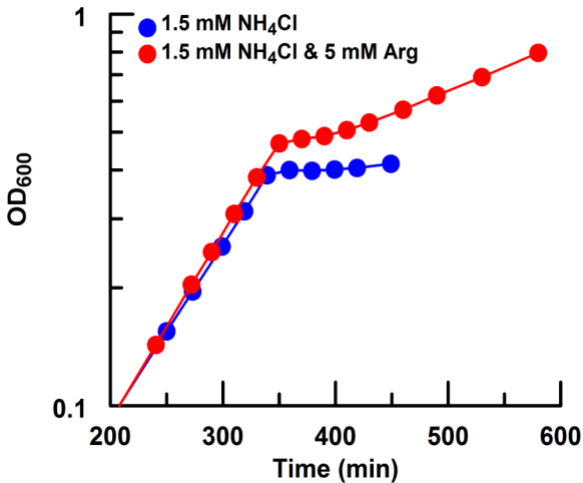

Supplement: Figure S2 — Nitrogen diauxie. The wild-type strain (PKUW13) was grown in minimal medium with 1.5 ammonium (blue circle) or 1.5 mM ammonium plus 5 mM arginine (red circle) as the nitrogen source. Download [file mbo004162913sf2.pdf]

**A**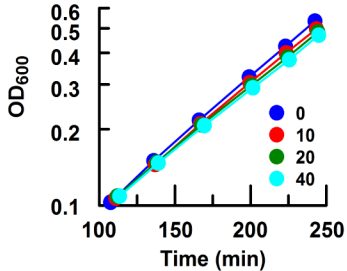**B**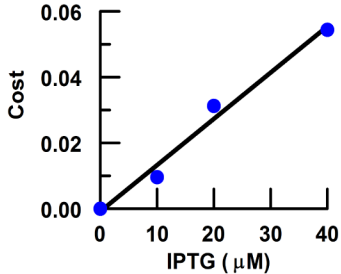

Supplement: Figure S3 — Full induction of the TC components results in a cost burden. (A) The TCE-Arg strain (PKUW81) was grown with 20 mM ammonium as the sole nitrogen source. Different concentrations (quantified in micromoles) of IPTG were supplemented to induce the expression of the arginine TC components. (B) The cost of growth of the TCE-Arg strain (PKUW81) with 20 mM ammonium as the sole nitrogen source. To calculate the cost of growth at different concentrations of IPTG, the wild-type strain (PKUW13) was grown in the same media as the TCE-Arg strain (PKUW81). Although different concentrations of IPTG had little effect on the growth rate of the wild-type strain, to exclude effects other than full induction of the TC components, the growth rate of the TCE strain was divided by the growth rate of the wild-type strain at the corresponding IPTG concentration. This normalized value was used to calculate the full induction cost of the TC components. Download [file mbo004162913sf3.pdf]

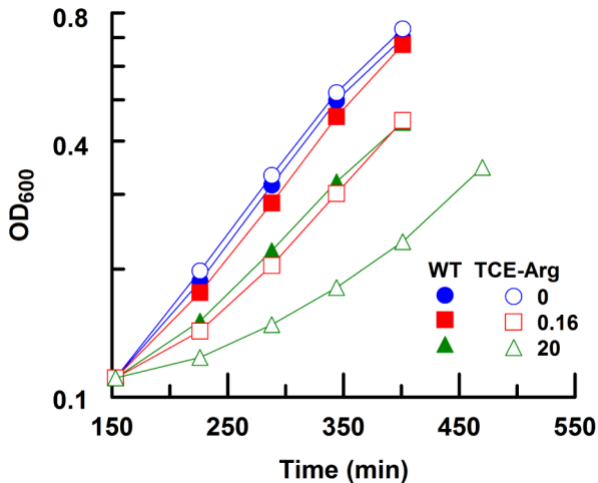

Supplement: Figure S4 — The TCE-Arg strain is more sensitive to l-canavanine. Both the wild-type strain (WT; closed symbols) and the TCE-Arg strain (open symbols) were grown in minimal medium with 40 µM IPTG and 20 mM NH4Cl as the nitrogen source. Different concentrations (0, 0.16, and 20 µg/ml) of l-canavanine were added to the cultures as indicated. Download [file mbo004162913sf4.pdf]

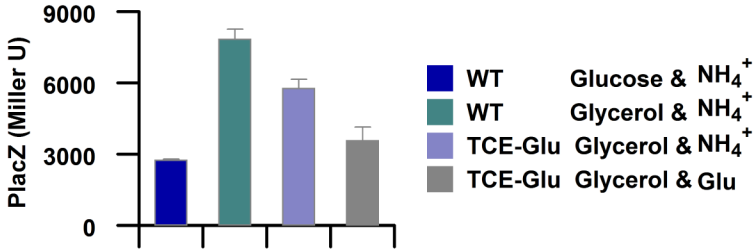

Supplement: Figure S5 — cAMP signaling in TCE-Glu and wild-type (WT) strains determined by analyzing the expression of the native lacZ gene. To fully deactivate LacI, 1 mM IPTG was added to both the precultures and the growth cultures. Data are expressed as means ± SD. Download [file mbo004162913sf5.pdf]
