# Supplementary material for: Deciphering the Principles of Bacterial Nitrogen Dietary Preferences: a Strategy for Nutrient Containment
Source: mBio. 2016 Jul 19;7(4):e00792-16. doi: 10.1128/mBio.00792-16 (PMC4958250; doi:10.1128/mBio.00792-16)
Supplement: Table S1 — Cost/benefit when glutamate was used as the sole nitrogen source. [file mbo004162913st1.pdf]

Table S1. Cost /benefit when glutamate was used as the sole nitrogen source

Strain[a]	WT	TCE-Glu[b]
Cost (-)/Benefit[c]	-0.76	0.27

[a] The strain symbol is the same as that in Table 1.

[b] 40 μM IPTG was supplemented to induce the expression of glutamate transport systems in the TCE-Glu strain (PKUW151).

[c] Cost/Benefit=$(\mu-\mu_0)/\mu_0$, where μ is the growth rate of WT or TCE strain with 20 mM glutamate as the sole nitrogen source, and μ_0 is the growth rate of WT with 20 mM ammonium as the sole nitrogen source.
