# Supplementary material for: Deciphering the Principles of Bacterial Nitrogen Dietary Preferences: a Strategy for Nutrient Containment
Source: mBio. 2016 Jul 19;7(4):e00792-16. doi: 10.1128/mBio.00792-16 (PMC4958250; doi:10.1128/mBio.00792-16)
Supplement: Table S2 — Growth rates of strains with arginine as the nitrogen source. [file mbo004162913st2.pdf]

Table S2. Growth rates of strains with arginine as nitrogen source

Genotype<br>Name	Wild-type<br>PKUW13	*amtB*<br>PKUW19	*glnK*<br>PKUW23	*glnKamtB*<br>PKUW15
Growth rate μ (hr^{-1})	0.16 ± 0.01	0.23 ± 0.01	0.37 ± 0.02	0.42 ± 0.02
