# Supplementary material for: Deciphering the Principles of Bacterial Nitrogen Dietary Preferences: a Strategy for Nutrient Containment
Source: mBio. 2016 Jul 19;7(4):e00792-16. doi: 10.1128/mBio.00792-16 (PMC4958250; doi:10.1128/mBio.00792-16)
Supplement: Text S1 — Supplemental text. Download [file mbo004162913s1.pdf]

Deciphering the principles of bacterial nitrogen dietary preferences: a strategy for nutrient containment

Jilong Wang[1,2], Dalai Yan[3], Ray Dixon [4] and Yi-Ping Wang[1,2]

Text S1

Potential polar effects of the *glnK* deletion on *amtB*

The ammonium transport *amtB* gene is located in an operon downstream of *glnK*. Even though the *glnK* deletion is in-frame (1), there could still be polar effects on *amtB* and it could be argued that the growth phenotype of the *glnK* strain is associated with altered expression of *amtB*. To demonstrate that the growth phenotype of the *glnK* deletion predominates over expression of *amtB*, we constructed *amtB* deletion strains and examined growth rates when arginine was used as nitrogen source (Table S2). We found that the growth rate of the *amtB* strain (PKUW19) increased by ~1.4-fold compared to the wild-type strain (PKUW13). However, the growth rate of the *glnK* strain (PKUW23) was ~2.3-fold faster than that of the wild-type strain, compared with a growth rate increase of ~2.6 fold for the *glnKamtB* double deletion strain (PKUW15) (Table S2). We also grew these strains with glutamate as the sole nitrogen source, and obtained similar results. Thus GlnK plays a major role in inhibiting exponential growth rather than AmtB and the growth phenotype of the *glnK* mutant is unlikely to be the consequence of a polar effect on *amtB*.

Supplementary References

1. **Baba T, Ara T, Hasegawa M, Takai Y, Okumura Y, Baba M, Datsenko KA, Tomita M, Wanner BL, Mori H.** 2006. Construction of *Escherichia coli* K-12 in-frame, single-gene knockout mutants: the Keio collection. Mol Syst Biol **2:**2006.0008.
